# Peroxiredoxin-1 Overexpression Attenuates Doxorubicin-Induced Cardiotoxicity by Inhibiting Oxidative Stress and Cardiomyocyte Apoptosis

**DOI:** 10.1155/2020/2405135

**Published:** 2020-07-29

**Authors:** Lai Jiang, Yanping Gong, Yida Hu, Yangyang You, Jiawu Wang, Zhetao Zhang, Zeyuan Wei, Chaoliang Tang

**Affiliations:** ^1^Department of Obstetrics and Gynecology, The First Affiliated Hospital of USTC, Division of Life Sciences and Medicine, University of Science and Technology of China, Hefei, Anhui 230001, China; ^2^Department of Anesthesiology, Renmin Hospital of Wuhan University, Wuhan, Hubei 430060, China; ^3^Department of Cardiology, The First Affiliated Hospital of USTC, Division of Life Sciences and Medicine, University of Science and Technology of China, Hefei, Anhui 230001, China; ^4^Department of Anesthesiology, The First Affiliated Hospital of USTC, Division of Life Sciences and Medicine, University of Science and Technology of China, Hefei, Anhui 230001, China; ^5^Department of Pharmacy, The First Affiliated Hospital of USTC, Division of Life Sciences and Medicine, University of Science and Technology of China, Hefei, Anhui 230036, China

## Abstract

*Background*. Previous research has shown that peroxiredoxin 1 (Prdx1) is an important modulator of physiological and pathophysiological cardiovascular events. This study is aimed at investigating the role and underlying mechanism of Prdx1 in doxorubicin- (DOX-) induced cardiotoxicity. Cardiac-specific expression of Prdx1 was induced in mice, and the mice received a single dose of DOX (15 mg/kg) to generate cardiotoxicity. First, our study demonstrated that Prdx1 expression was upregulated in the heart and in cardiomyocytes after DOX treatment. Second, we provided direct evidence that Prdx1 overexpression ameliorated DOX-induced cardiotoxicity by attenuating oxidative stress and cardiomyocyte apoptosis. Mechanistically, we found that DOX treatment increased the phosphorylation level of apoptosis signal-regulating kinase-1 (ASK1) and the downstream protein p38 in the heart and in cardiomyocytes, and these effects were decreased by Prdx1 overexpression. In contrast, inhibiting Prdx1 promoted DOX-induced cardiac injury via the ASK1/p38 pathway. These results suggest that Prdx1 may be an effective therapeutic option to prevent DOX-induced cardiotoxicity.

## 1. Introduction

Doxorubicin (DOX), an anthracycline antibiotic, is a broad-spectrum antitumor drug that is widely used in the clinical treatment of various malignant tumors [1, 2]. Studies have confirmed that cardiomyocytes have a strong affinity for DOX, and DOX easily accumulates in cardiomyocytes, leading to severe cardiotoxicity [3, 4]. DOX-induced cardiotoxicity usually occurs when the cumulative amount is more than 500 mg/m^2^. However, recent studies show that 21% of patients have treatment-related cardiotoxicity when the cumulative amount is less than 300 mg/m^2^ [5, 6]. Multiple molecular mechanisms have been reported to contribute to the processes of DOX-induced myocardial injury, including oxidative stress, the inflammatory response, mitochondrial dysfunction, and cardiomyocyte apoptosis [7, 8]. Accordingly, targeting molecules or genes associated with the above processes represents a therapeutic opportunity for treating DOX-induced cardiotoxicity.

Peroxiredoxins (Prdxs) are a ubiquitous family of antioxidant enzymes and include six isoforms of peroxidases (Prdx1-Prdx6), which can catalyze the reduction of peroxynitrite, hydrogen peroxide, and other hydroperoxides [9, 10]. Prdxs have also been identified as regulators of redox-sensitive signaling [11, 12]. Among them, Prdx1 is well known for its protective roles in many diseases, such as aging, acute tissue injury, neurodegenerative diseases, and cancers, by scavenging reactive oxygen species (ROS) [9, 13–15]. Lv et al. reported that Prdx1 knockout increased the ROS content and reduced the antioxidant capacity in the lung after lipopolysaccharide treatment [16]. Mei et al. reported that Prdx1 overexpression inhibits oxidative stress and apoptosis in renal tubulointerstitial fibrosis [17].

There is substantial research indicating that Prdx1 is an important modulator of various cardiovascular events [18, 19]. However, whether Prdx1 plays roles in DOX-induced cardiotoxicity remains unclear. In this study, we report for the first time that Prdx1 expression was upregulated in the hearts and cardiomyocytes after DOX treatment. Prdx1 overexpression ameliorated DOX-induced cardiac dysfunction and injury potentially by inhibiting oxidative stress and cardiomyocyte apoptosis. Collectively, our results suggest that Prdx1 may be an effective therapeutic option to prevent DOX-induced cardiotoxicity.

## 2. Materials and Methods

### 2.1. Animal Experiments

All procedures involving animals were conducted according to the NIH *Guide for the Care and Use of Laboratory Animals* and were approved by the Ethics Committee of Renmin Hospital of Wuhan University. Male C57BL/6J mice (25.5 ± 2 g, 8-10 weeks) were purchased from Beijing Vital River Laboratory Animal Technology Co., Ltd. (Beijing, China). To specifically overexpress Prdx1 in the myocardium, randomly chosen mice were injected in the heart with either AAV9-Prdx1 or AAV9-negative control (AAV9-NC) at a dose of 1 × 10^11^ viral particles per mouse [20]. AAV9-NC and AAV9-Prdx1 were generated by Shanghai Genechem Co., Ltd. (Shanghai, China). Four weeks after AAV9 injection, the animals received a single dose of DOX (15 mg/kg, i.p.) or an equal volume of normal saline (NS) as described previously [21]. All animals were observed daily and weighed two days after DOX insult.

### 2.2. Echocardiography and Hemodynamics

Eight days after DOX insult, transthoracic echocardiography was performed to assess the heart function of the animals. Left ventricular (LV) geometry was assessed with both parasternal short-axis and long-axis views at the midpapillary muscle level. In addition, invasive hemodynamic monitoring was performed with a Millar Pressure-Volume System (Millar Instruments, USA).

### 2.3. Biochemical Determination

After echocardiography and hemodynamic analysis, blood specimens were obtained and centrifuged at 3000 *g* at 4°C for 15 min to separate serum. In addition, cardiac tissues were removed and homogenized in ice-cold phosphate-buffered saline (PBS). Serum and cardiac tissue concentrations of lactate dehydrogenase (LDH) and creatine kinase isoenzymes (CK-MB) were measured with commercially available kits (Nanjing Jiancheng Bioengineering Institute, China).

### 2.4. Histological Analysis

At the end of the experiment, the cardiac tissues were removed, immersed in 10% formalin, and then embedded in paraffin. Subsequently, the cardiac tissues were cut into 5 *μ*m slides and stained with hematoxylin and eosin (H&E). The semiquantitative grade of cardiomyocyte degeneration in each section was analyzed as described previously [22]. In addition, terminal deoxynucleotidyl transferase dUTP nick-end labeling (TUNEL) staining was performed to detect cardiomyocyte apoptosis according to the manufacturer's instructions (Roche). All images were analyzed with Image-Pro plus 6.0 software.

### 2.5. Cell Culture and Treatments

Neonatal rat ventricular myocytes (NRVMs) were isolated from Sprague-Dawley rats within 3 days of birth as previously described [23]. The NRVMs were cultured in Dulbecco's modified Eagle's medium and treated with DOX (1 *μ*mol/L) for 12 h [24]. To overexpress Prdx1 in vitro, NRVMs were preincubated with adenoviruses carrying Prdx1 (Ad-Prdx1; Vigene Bioscience, Jinan, China) or an NC sequence (Ad-NC) for 12 h and then treated with DOX for 12 h. Adenanthin, a diterpenoid isolated from the leaves of Isodon adenanthus, has been shown to inhibit the enzymatic activity of Prdx1 by targeting its conserved resolving cysteines [25, 26]. Thus, we used adenanthin to further clarify the role of Prdx1 in DOX-mediated cardiotoxicity.

### 2.6. Measurement of Oxidative Stress Level

Dihydroethidium (DHE, Sigma-Aldrich) staining was performed according to manufacturer's instructions. Briefly, frozen heart sections were incubated with 10 *μ*mol/L DHE in a humidified and light-protected chamber for 20 min at 37°C. The sections were examined under a fluorescence microscope (Olympus, Japan) and analyzed using Image-Pro plus 6.0 software. To further assess oxidative stress levels, the fresh hearts were homogenized and then centrifuged at 3000 rpm at 4°C for 10 min to collect the supernatant fractions. The activity of total superoxide dismutase (SOD), catalase (CAT), and NADPH oxidase, and the concentration of malondialdehyde (MDA) were measured in myocardial tissue and NRVMs using commercially available kits (Beyotime Biotechnology, China).

### 2.7. Western Blot Analysis

Total protein was extracted from LV tissue or NRVMs with RIPA buffer, and the protein concentration was detected with a BCA assay kit. The protein samples were fractionated via SDS-polyacrylamide gel electrophoresis and transferred onto polyvinylidene fluoride (PVDF) membranes (Millipore, China). Subsequently, the blots were blocked with 5% nonfat milk and incubated with primary antibodies, including Prdx1 (1 : 1000; Abcam), Bax (1 : 1000; Abcam), Bcl-2 (1 : 1000; Abcam), c-caspase-3 (1 : 1000; Abcam), p-p38 (1 : 1000; Cell Signaling Technology), p38 (1 : 1000; Cell Signaling Technology), apoptosis signal-regulating kinase-1 (ASK1, 1 : 1000; Cell Signaling Technology), p-ASK1 (1 : 1000; Cell Signaling Technology), and GAPDH (1 : 1000; Abcam). Then, the blots were incubated with horseradish peroxidase- (HRP-) conjugated secondary antibody (1 : 10000; Abcam) for 1 h, and protein expression was analyzed by an Odyssey infrared imaging system (LI-COR Biosciences).

### 2.8. Quantitative Real-Time PCR (qRT-PCR)

Total RNA was extracted from LV tissues or NRVMs and then quantified by spectrophotometry. After reverse transcription into cDNA, quantitative analysis was performed using a LightCycler 480 thermal cycler (Roche, Germany). PCR amplification was performed as follows: 95°C for 5 min, followed by 39 cycles of 95°C for 10 s, 60°C for 20 s, and 72°C for 20 s. The mRNA expression of target genes was analyzed with the 2^-*ΔΔ*Ct^ method and normalized to GAPDH gene expression. All details of the PCR primer sequences are presented in Table 1.

### 2.9. Statistical Analysis

The results in this study are expressed as the mean ± standard deviation. Differences between two groups were determined by unpaired Student's *t*-tests. Differences among multiple groups were compared by one-way analysis of variance followed by a post hoc Tukey test. *P* < 0.05 was considered indicative of a statistically significant difference.

## 3. Results

### 3.1. Prdx1 Expression Is Increased in the Heart and in Cardiomyocytes after DOX Treatment

**Figure 1:**
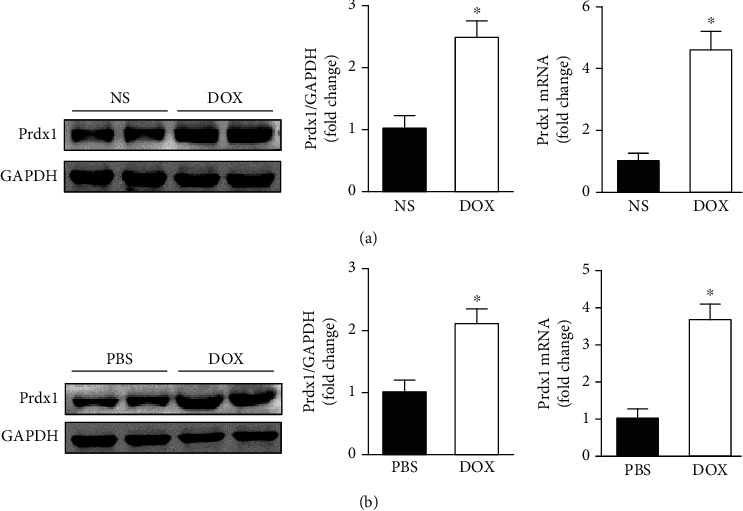
Prdx1 expression is increased in the heart and in cardiomyocytes after DOX treatment. (a) The protein and mRNA expression of Prdx1 in the heart 8 days after DOX treatment (*n* = 4, ^∗^*P* < 0.05 compared with the NS group). (b) The protein and mRNA expression of Prdx1 in cardiomyocytes treated with DOX (*n* = 4, ^∗^*P* < 0.05 compared with the PBS group).

We first determined Prdx1 expression in the heart and in cardiomyocytes after DOX treatment. Western blotting and RT-PCR analyses showed that Prdx1 mRNA and protein expressions were increased in the heart 8 days after DOX treatment (Figure 1(a)). In addition, Prdx1 mRNA and protein expression levels were increased in NRVMs after DOX incubation (Figure 1(b)). Taken together, these results suggest that Prdx1 may be implicated in DOX-induced cardiac injury.

### 3.2. Prdx1 Overexpression Protected against Cardiac Injury after DOX Treatment in Mice

**Figure 2:**
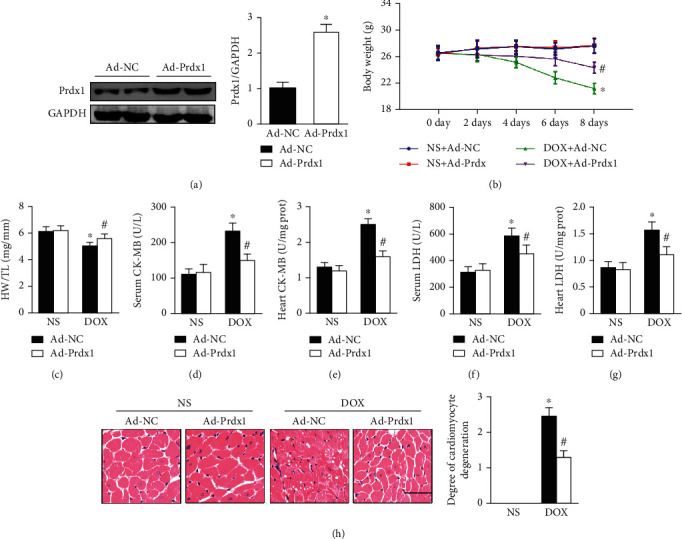
Prdx1 overexpression protected against cardiac injury induced by DOX. (a) The protein levels of Prdx1 four weeks after AAV9-Prdx1 injection in mice (*n* = 4). (b, c) The results of body weight and HW/TL ratio measurements in mice (*n* = 6). (d–g) Biochemical determination of CK-MB and LDH levels in the heart and serum for the indicated groups (*n* = 6). (h) HE staining shows the pathological structure of the heart in mice (*n* = 5). ^∗^*P* < 0.05 compared with the NS group; ^#^*P* < 0.05 compared with the DOX group.

The results showed that the body weights and heart weight/tibia length (HW/TL) ratios were significantly reduced after DOX treatment, and these effects were significantly ameliorated by Prdx1 overexpression (Figures 2(a)–2(c)). In addition, sensitive biomarkers for myocardial injury, including LDH and CK-MB, were increased in the serum and heart after DOX administration, and these effects were significantly attenuated by Prdx1 overexpression (Figures 2(d)–2(g)). Histological examination revealed that Prdx1 overexpression decreased DOX-induced cardiomyocyte vacuoles and degeneration (Figure 2(h)).

### 3.3. Prdx1 Overexpression Ameliorated Cardiac Dysfunction Induced by DOX in Mice

**Figure 3:**
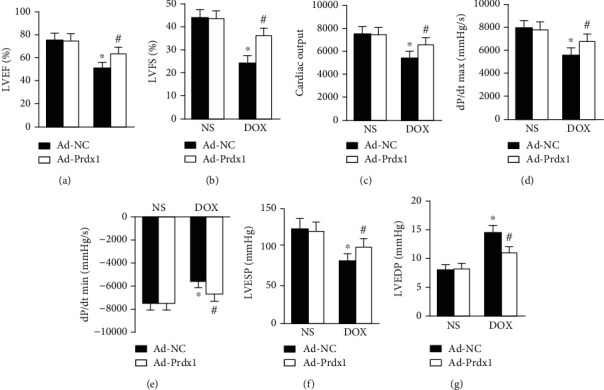
Prdx1 overexpression attenuated cardiac dysfunction induced by DOX. (a, b) Echocardiographic parameters for each group (*n* = 8). (c–g) Hemodynamic parameters for each group (*n* = 6). ^∗^*P* < 0.05 compared with the NS group; ^#^*P* < 0.05 compared with the DOX group.

After DOX treatment, the animals exhibited cardiac dysfunction as indicated by reduced LV ejection fraction (LVEF) and fractional shortening (LVFS) values, as reported previously [27]. However, Prdx1 overexpression improved these parameters in DOX-treated mice (Figures 3(a) and 3(b)). In addition, DOX-induced LV systolic and diastolic dysfunction was markedly ameliorated by Prdx1 overexpression (Figures 3(c)–3(g)).

### 3.4. Prdx1 Overexpression Reduced Oxidative Stress Induced by DOX in Mice

**Figure 4:**
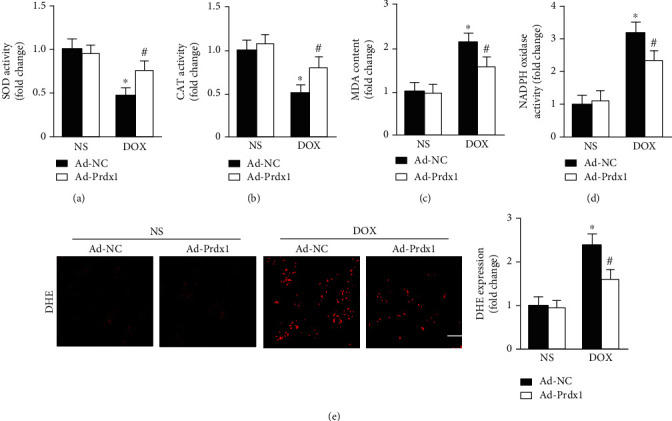
Prdx1 overexpression attenuated oxidative stress induced by DOX. (a–d) Quantitative results for SOD, CAT, NADPH oxidase activity, and MDA content in the hearts of mice (*n* = 6). (e) Representative DHE staining images of the heart (*n* = 5). ^∗^*P* < 0.05 compared with the NS group; ^#^*P* < 0.05 compared with the DOX group.

The results showed that DOX insult caused significant oxidative stress in mice as evidenced by decreased SOD and CAT activity and increased MDA content and NADPH oxidase activity (Figures 4(a)–4(d)). Interestingly, Prdx1 overexpression preserved SOD and CAT activity and reduced MDA content and NADPH oxidase activity (Figures 4(a)–4(d)). The DHE staining results also showed that Prdx1 overexpression significantly reduced oxidative stress levels in the heart (Figure 4(e)).

### 3.5. Prdx1 Overexpression Protected against Cardiomyocyte Apoptosis Induced by DOX in Mice

**Figure 5:**
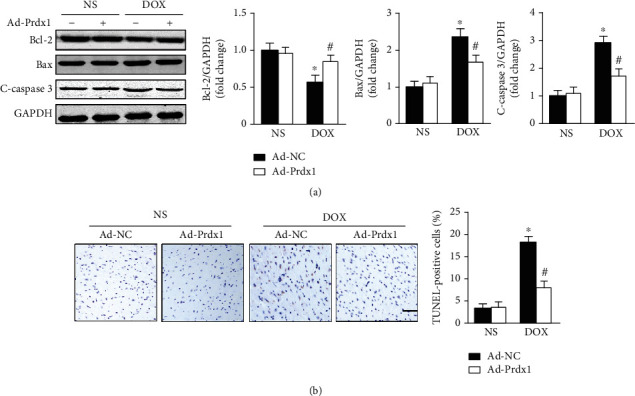
Prdx1 overexpression attenuated cardiomyocyte apoptosis induced by DOX. (a) Western blots showing the Bcl-2, Bax, and c-caspase3 levels in the hearts of mice (*n* = 4). (b) Representative TUNEL staining images of the heart (*n* = 5). ^∗^*P* < 0.05 compared with the NS group; ^#^*P* < 0.05 compared with the DOX group.

The results showed that DOX treatment significantly increased the expression of Bax and cleaved caspase-3 and decreased Bcl-2 levels (Figure 5(a)). However, Prdx1 overexpression alleviated the effect of DOX on these proteins (Figure 5(a)). The TUNEL staining results further confirmed that Prdx1 overexpression alleviated DOX-induced cardiomyocyte apoptosis in mice (Figure 5(b)).

### 3.6. Prdx1 Overexpression Relieved DOX-Induced Cardiotoxicity In Vitro

**Figure 6:**
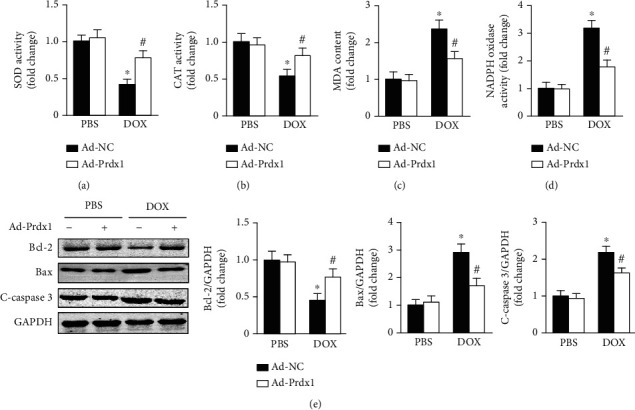
Prdx1 overexpression relieves DOX-induced cardiotoxicity in vitro. (a–d) Quantitative results for SOD, CAT, NADPH oxidase activity, and MDA content in each group (*n* = 6). (e) Western blots showing the Bcl-2, Bax, and c-caspase3 levels in each group (*n* = 4). ^∗^*P* < 0.05 compared with the PBS group; ^#^*P* < 0.05 compared with the DOX group.

Consistent with the results of in vivo, we found that Prdx1 overexpression preserved SOD and CAT activity and decreased MDA content and NADPH oxidase activity after DOX treatment (Figures 6(a)–6(d)). In addition, Prdx1 overexpression significantly attenuated the decreased Bax and cleaved caspase-3 levels and improved Bcl-2 expression after DOX treatment (Figure 6(e)).

### 3.7. Prdx1 Overexpression Inhibited ASK1/p38 Pathway Activation

**Figure 7:**
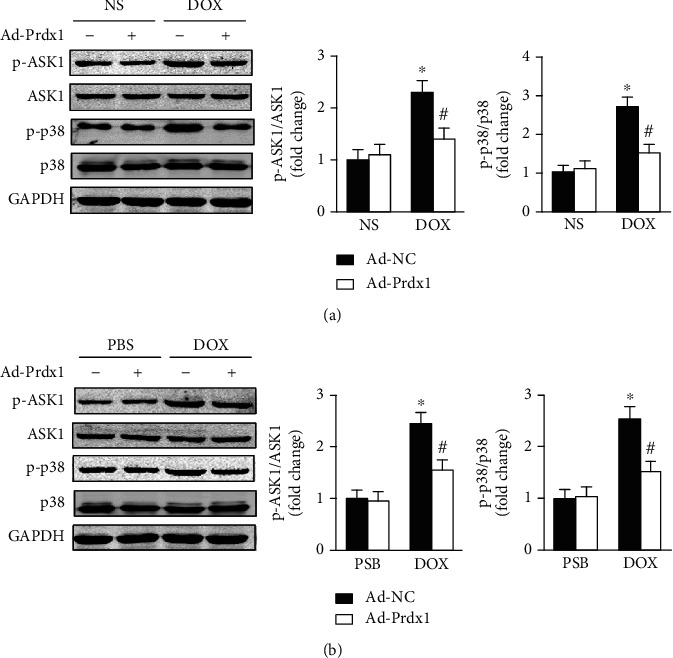
Prdx1 overexpression inhibits ASK1/p38 pathway activation. (a) Western blots showing the p-ASK1, ASK1, p-p38, and p38 levels in the hearts of mice (*n* = 4). (b) Western blots showing the p-ASK1, ASK1, p-p38, and p38 levels in NVRMs (*n* = 4). ^∗^*P* < 0.05 compared with the NS or PBS group; ^#^*P* < 0.05 compared with the DOX group.

ASK1, a key redox-sensitive protein kinase, is a critical regulator of oxidative damage and cell apoptosis [28, 29]. Thus, we investigated whether the protective effects of Prdx1 are associated with the ASK1/p38 pathway. Consistent with previous research [30], DOX treatment upregulated the phosphorylation levels of ASK1 and p38 in the heart, while Prdx1 overexpression downregulated ASK1 and p38 phosphorylation levels (Figure 7(a)). In addition, the results also showed that Prdx1 overexpression decreased ASK1 and p38 phosphorylation levels after DOX treatment in vitro (Figure 7(b)).

### 3.8. Prdx1 Inhibition Exacerbated DOX-Induced Cardiotoxicity

**Figure 8:**
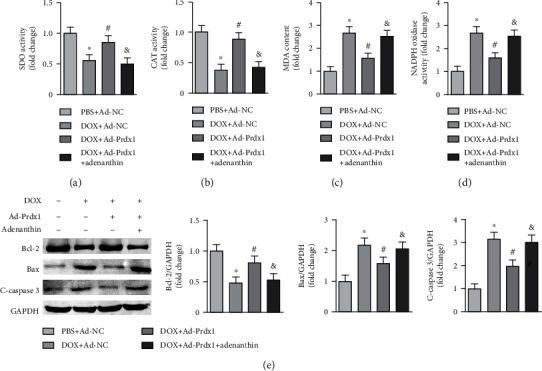
Prdx1 inhibition exacerbated DOX-induced cardiotoxicity. (a–d) Quantitative results for SOD, CAT, NADPH oxidase activity, and MDA content in each group (*n* = 6). (e) Western blots showing the Bcl-2, Bax, and c-caspase3 levels in each group (*n* = 4). ^∗^*P* < 0.05 compared with the PBS+Ad-NC group; ^#^*P* < 0.05 compared with the DOX+Ad-NC group; ^&^*P* < 0.05 compared with the DOX+Ad-Prdx1 group.

The results showed that adenanthin, a Prdx1-specific inhibitor, almost completely eliminated the antioxidative stress effects of Prdx1 by decreasing SOD and CAT activity and increasing MDA content and NADPH oxidase activity (Figures 8(a)–8(d)). In addition, the results also showed that Prdx1 overexpression downregulated the expression of Bax and cleaved caspase-3 and improved Bcl-2 levels after DOX treatment, but adenanthin could counteract this effect (Figure 8(e)).

## 4. Discussion

In the present study, we demonstrated the protective effect of Prdx1 against DOX-induced cardiotoxicity and clarified the potential molecular mechanisms. First, our study demonstrated that Prdx1 expression was upregulated in the heart and in cardiomyocytes after DOX insult. Second, we provided direct evidence that Prdx1 overexpression ameliorated DOX-induced cardiotoxicity by attenuating oxidative stress and cardiomyocyte apoptosis. Mechanistically, we found that DOX treatment increased ASK1 and p38 phosphorylation levels in the heart and in cardiomyocytes, but these effects were decreased by Prdx1 overexpression. In contrast, inhibiting Prdx1 promoted DOX-induced cardiotoxicity via the ASK1/p38 pathway. Based on these findings, we speculated that Prdx1 might be exploited as a potential therapeutic target for DOX-induced cardiotoxicity.

Prdx1 was discovered twenty years ago and is well known as an antioxidant and redox signaling protein. Prdx1 is expressed in the cytosol of many types of cells and tissues and plays an important role in maintaining redox balance [31, 32]. Notably, increasing evidence has shown that Prdx1 plays important roles in various cardiovascular diseases. Previous research has shown that Prdx1 deficiency induces excessive oxidative stress and increases the number of macrophage foam cells in atherosclerotic plaques in mice [33, 34]. Another study also showed that Prdx1 overexpression alleviates cardiomyocyte apoptosis by scavenging ROS during myocardial ischemia/reperfusion injury [35]. However, the role of Prdx1 in DOX-induced cardiotoxicity remains unclear. Our study demonstrated that Prdx1 expression was upregulated in the DOX-treated hearts and cardiomyocytes, suggesting that Prdx1 might be implicated in the cardiac injury response to DOX. In addition, AAV9-Prdx1 was injected to further detect the effects of Prdx1 in the heart of mice. The results showed that Prdx1 overexpression ameliorated DOX-induced cardiac dysfunction and structural damage, indicating a protective effect of Prdx1 on DOX-mediated cardiotoxicity.

Oxidative stress, which is an imbalance between ROS and the antioxidant system, plays a pivotal role in multiple cardiovascular diseases [36, 37]. ROS represent a family of oxygen-containing molecules, including superoxide, hydroxyl radicals, peroxynitrite, and nonradicals, such as hydrogen peroxide [36, 38]. To counter excessive ROS accumulation and maintain redox homeostasis, cells are equipped with antioxidant defense systems, including enzymatic antioxidants (such as SOD and CAT) and nonenzymatic antioxidants (such as glutathione) [36, 39]. To date, a large number of studies have clearly demonstrated that increased ROS accumulation and decreased antioxidant enzymes or proteins are important pathological features in DOX-induced cardiac injury and dysfunction [8, 40]. As a result, suppressing oxidative stress and scavenging ROS are beneficial in the treatment of DOX-induced cardiotoxicity.

Previous research has shown that Prdx1 is an antioxidant enzyme and can catalyze the reduction of peroxides and peroxynitrite [10, 32]. Thus, we investigated whether Prdx1 affects oxidative stress in DOX-induced cardiotoxicity. The results showed that Prdx1 overexpression markedly increased SOD and CAT activity and decreased MDA content and NADPH oxidase activity in the hearts of DOX-treated mice. In addition, DHE staining results also showed that Prdx1 overexpression markedly reduced oxidative stress levels in the hearts of DOX-treated mice. Consistent with the in vivo results, we also found that Prdx1 overexpression relieved DOX-induced oxidative stress in vitro. We further investigated the potential molecular mechanisms of Prdx1 in DOX-induced cardiotoxicity.

Among the potential mechanisms that have been studied by many researchers, apoptosis is an important etiology and pathological process in DOX-induced cardiac injury [41, 42]. Previous studies showed that DOX exposure at submicromolar concentrations caused apoptotic cell death in cardiomyocytes, and oxidative stress has been shown to be responsible for cardiac injury and cardiomyocyte apoptosis [43, 44]. To detect the role of Prdx1 in DOX-induced cardiomyocyte apoptosis, western blotting and TUNEL staining were performed. The results showed that DOX treatment resulted in significantly reduced Bcl-2 expression, augmented Bax and C-caspase3 levels, and more TUNEL-positive cells in the heart. These effects were abrogated by Prdx1 overexpression. In addition, the results further showed that DOX-induced cardiomyocyte apoptosis was prevented by Prdx1 overexpression in cell experiments in vitro. This study further indicated that Prdx1 alleviates DOX-induced cardiac injury by inhibiting cardiomyocyte apoptosis.

ASK1, a member of the mitogen-activated protein kinase kinase kinase (MAP3K) family, is a key redox-sensitive protein kinase and is responsive to stress-induced cell damage that activates its downstream signaling cascades [45, 46]. Previous studies have shown that the ASK1/p38 signaling pathway plays an important role in oxidative stress and cell apoptosis, which is regulated by Prdx1 [47, 48]. Lu et al. reported that Prdx1 overexpression suppresses oxidative stress and neuronal apoptosis by decreasing ASK/p38 phosphorylation in a mouse model of subarachnoid hemorrhage [49]. Guo et al. also reported that Prdx1 silencing aggravates ROS generation and cardiomyocyte apoptosis through p38 activation during myocardial ischemia/reperfusion injury [35]. However, it is not clear whether Prdx1 inhibits DOX-induced cardiotoxicity and is associated with the ASK/p38 signaling pathway. In this study, our data showed that DOX treatment increased the phosphorylation levels of ASK1 and p38 in the heart and in cardiomyocytes, which is consistent with previous studies. Interestingly, these effects were abrogated by Prdx1 overexpression, indicating that the cardioprotective effect of Prdx1 may be associated with the ASK1/p38 pathway.

**Figure 9:**
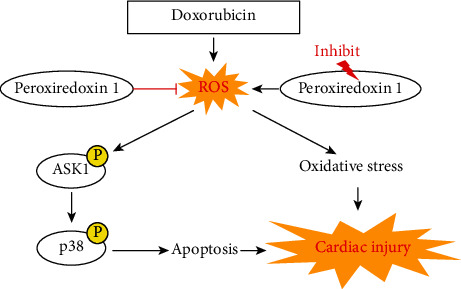
Peroxiredoxin-1 overexpression attenuates doxorubicin-induced cardiotoxicity by inhibiting oxidative stress and cardiomyocyte apoptosis.

In conclusion, we have provided novel insights into the roles of Prdx1 in regulating DOX-induced cardiac injury and dysfunction. The results showed that Prdx1 ameliorated DOX-induced cardiotoxicity by inhibiting oxidative stress and cardiomyocyte apoptosis (Figure 9).

## Figures and Tables

**Table 1:** Primer sequences for RT-PCR assays.

Gene	Species	Sequence (5′-3′)	
Prdx1	Mouse	Forward	AATGCAAAAATTGGGTATCCTGC
		Reverse	CGTGGGACACACAAAAGTAAAGT

Prdx1	Rat	Forward	TTTGTGTGTCCCACGGAGAT
		Reverse	TAATGGTGCGCTTGGGATCTG

GAPDH	Mouse	Forward	AACTTTGGCATTGTGGAAGG
		Reverse	CACATTGGGGGTAGGAACAC

GAPDH	Rat	Forward	GACATGCCGCCTGGAGAAAC
		Reverse	AGCCCAGGATGCCCTTTAGT

## Data Availability

We declare that the materials described in the manuscript, including all relevant raw data, will be freely available to any scientist wishing to use them for noncommercial purposes, without breaching participant confidentiality.
